# Should we aim for genetic improvement in host resistance or tolerance to infectious pathogens?

**DOI:** 10.3389/fgene.2012.00272

**Published:** 2012-12-14

**Authors:** Andrea B. Doeschl-Wilson, Ilias Kyriazakis

**Affiliations:** ^1^Genetics and Genomics, The Roslin Institute and R(D)SVS of the University of EdinburghRoslin, UK; ^2^Department of Agriculture, Food, and Rural Development, Newcastle UniversityNewcastle upon Tyne, UK

There is an imperative to consider alternative strategies to pharmaceuticals to control infectious diseases amongst livestock. Recent advances in genetics and genomics have highlighted the potential for genetic control strategies to maintain high health and performance levels in livestock populations. This special research topic focuses on two alternative host defence strategies for coping with infectious pathogens that could be tackled for genetic improvement: **host resistance** vs. **host tolerance**. Resistance refers to mechanisms that restrict the reproduction rate of pathogens within a host, e.g., by blocking pathogen entry or limiting pathogen replication. Tolerance, in contrast, refers to the ability of a host to limit the detrimental impact pathogens can inflict on host performance (e.g., growth, milk/egg production, and fertility), without affecting pathogen burden *per se*. Tolerance captures all immune mechanisms that are not directly related to the reduction of pathogen burden, such as damage prevention or repair, as well as mechanisms that regulate self-harm inflicted by immune response components.

In contrast to the rapid expansion of identified genetic loci associated with host resistance, information of genetic loci or pathways associated with tolerance mechanisms is extremely sparse. However, a deeper understanding of the genetic control underlying both mechanisms is important in order to decide upon disease control strategies and avoid undesirable outcomes of genetic improvement programmes. This is firstly because at genetic level, resistance and tolerance may be antagonistically related. Secondly, although resistance and tolerance may have a similar impact on individual health and performance, they can have contrasting effects on performance outcomes and on disease prevalence at a population level. In particular, whilst improving host resistance could lead to disease eradication, this is unlikely if hosts are tolerant, as they can harbor the pathogen without showing obvious symptoms. On the other hand, it has been argued that increasing host resistance (but not tolerance) would fuel the arms race between host and pathogen, and stimulate pathogen evolution toward higher virulence. Furthermore, whereas disease resistance mechanisms are often pathogen-specific (e.g., mobilization of specific immune cells), tolerance mechanisms that prevent or repair damage may be more host than pathogen specific, and may thus offer generic protection for a range of pathogens. Hence, changing tolerance may be more beneficial in situations where individuals are exposed to a variety of pathogens or pathogen strains (as is the case in many commercial farms), with high risk of pathogen evolution, and where disease eradication has proven difficult (e.g., if asymptomatic carriers are present).

In contrast to evolutionary biology and plant breeding, livestock breeding has only recently started to appreciate the importance of distinguishing between resistance and tolerance to pathogens and to study their relationship and implications. This special research topic draws together animal scientists with expertise in molecular and quantitative genetics, immunology, epidemiology, evolutionary biology, and mathematical modeling to address the question “Should we aim for genetic improvement of host resistance or host tolerance to infectious pathogens” from different perspectives. The diverse contributions to this topic:

1. Provide an overview of the state-of-the-art understanding of resistance and tolerance in domestic livestock populations, with a focus on the application of genetic and genomic tools for host genetic improvement of either.
2. Lay out methodologies and data requirements for accurately quantifying resistance and tolerance for subsequent genetic studies.
3. Investigate the advantages and disadvantages of improving resistance vs. tolerance for specific relevant livestock diseases.

This special research topic kicks off with our own contribution that sets the stage for the development of the topic by the other authors (Doeschl-Wilson et al., 2012a,b). Hypothesizing that genetic improvement of host tolerance to infectious pathogens is first of all handicapped by difficulties in determining the tolerance phenotype, we then investigate what is needed to obtain accurate estimates of tolerance phenotypes. Our first article concentrates on group tolerance, which is the current state-of-the-art for quantifying tolerance, whereas the second article proposes new analytical solutions for extending the framework to the level of individuals. Complementary to this, Kause and Ødegård (2012) present recent statistical methods to estimate genetic parameters associated with tolerance and tolerance-related traits. Each method requires careful consideration of data requirements and underlying conditions for implementation into future breeding programmes.

Glass (2012) addresses resistance and tolerance from an immunological perspective at molecular level. Reviewing sequential immunological processes involved in the host response to infectious pathogens, she explores whether resistance and tolerance mechanisms are likely to be controlled by the same set of genes or molecular pathways, and proposes new avenues for identifying new resistance or tolerance genes. As resistance and tolerance constitute two alternative, resource-costly, host defence mechanisms, a trade-off between both strategies may occur when resources are limited. Rauw (2012) investigates origins and consequences of such trade-offs by considering resistance and tolerance from a resource allocation viewpoint.

As emphasized in this special topic and elsewhere, the relative merits of improving host resistance and tolerance require careful consideration and may differ between diseases and livestock populations. Looking at the wider implications of improving either trait, Bishop (2012) addresses under what circumstances tolerance may be worth considering as a breeding goal, and applies his theoretical framework to nematode infections in ruminants. Guy et al. (2012) review current perspectives on selective breeding for disease resistance and tolerance in pigs, with an emphasis on industry applications. Rowland et al. (2012) discuss recent evidence of host genetic control in the response of pigs to the Porcine Reproductive and Respiratory Syndrome (PRRS) virus, in the light of resistance and tolerance. PRRS is a high priority pig disease world-wide, and currently there is a lively debate on whether to improve resistance or tolerance. Looking at Salmonella and Campylobacter infections in poultry, Calenge and Beaumont (2012) present an example where host tolerance to infectious pathogens is undesirable, and review current knowledge about host genetic control of two distinct resistance mechanisms, i.e., resistance to intestinal colonization and resistance to bacterial persistence. Finally, Detilleux (2012) uses a mathematical modeling approach to investigate the implications of selection for improved resistance or tolerance on performance and health at the level of individuals and populations, when applied to bovine mastitis. Mathematical models have the advantage that traits which are difficult to measure in practice, such as tolerance, can be predicted. This enables the role of influencing factors to be assessed systematically.

We hope that this special research topic moves us a step forward in our understanding of these two important, highly complex traits associated with livestock health and production, and the development of sustainable genetic improvement strategies.
